# A Treatise on Inflammation

**Published:** 1839-01-01

**Authors:** 


### A Treatise on Inflammation. By *James Macartney*, M.D. F.R.S., &c. &c. &c. Quarto, pp. 214. Two Plates. Longman's, 1838.

It will probably appear a hazardous experiment to publish a work upon inflammation at present. The subject is so hackneyed as to excite little curiosity or attention, and few persons would conceive it possible that any man could advance new views of its nature, or new plans of any value in its management.

But Dr. Macartney's reputation will secure that notice which might otherwise be denied, and command a full audience and respectful attention whenever he addresses the profession. We may be satisfied that we shall meet with ingenuity and originality, and whether we are convinced or not, we are certain of pleasure and instruction.

Dr. Macartney tells us in a brief advertisement, that:—

"The present Treatise contains the theory and practice, respecting the subject of inflammation, which I have taught during many years in my lectures on surgery. Its publication did not become necessary, until I had resigned my professorship in the University of Dublin, as I had annually the best means, perhaps, of promulgating and explaining my views in the form of lectures. The delay has been attended with the great advantage, of enabling me to verify and improve my early opinions, by a longer experience, and by the successful practice of my pupils, who are now settled in every part of the United Kingdom and throughout our Colonies. Whatever the profession may think of the doctrines I have advanced on the subject of inflammation, the practice founded on them is at present established too extensively, and confirmed by the experience of too many individuals, to admit of controversy."

We shall run through these lectures, or this Essay, as rapidly as is consistent with justice both to the author and our readers. We shall content ourselves with noticing those parts that are either novel, or give some new turn or some striking confirmation to what has been familiar.

The work is divided into fourteen parts, or sections, in which Dr. Macartney discusses in succession—The History of Inflammation—Phenomena of Inflammation—The Real Consequences of Inflammation—The Reputed Consequences of Inflammation—The Different Modes of Reparation—Cicatrization—The Reparation of the Different Tissues—The Constitutional Causes of Inflammation—The Local Causes of Inflammation—The Proximate Cause of Inflammation—Species of Inflammation—Congestion as contradistinguished from Inflammation—The Remedies for Inflammation.

1. *History of Inflammation.*—Dr. Macartney presents a brief sketch of the different classes of animals, in reference to their liability to inflammation.

In those zoophytes which present no visible nerves, and a very simple nervous system, none of the phenomena of inflammation are exhibited.

Dr. Macartney alludes to the phenomena of reproduction in the articulata. The instances are numerous, the phenomena rather varied, and we do not perceive conclusive proof of the non-existence of inflammation in the class.

The class mollusca, says our author, do not seem to be capable of genuine inflammation.

Ascending to the vertebrata, Dr. Macartney does not believe it possible to produce the genuine effects of inflammation in either amphibia, or reptiles, or fishes.

"In conducting some experiments on the swimming-bag of *fishes*, I was surprised to find that the wounds made into the belly of the animals did not inflame. I was therefore curious to know what injuries fishes would bear without producing inflammation. Having taken some living fishes from the water, I introduced pieces of wire beneath the skin and amongst the muscles of the body; the fishes were then returned to the water, and on examining them several days afterwards, I found that no suppuration had taken place. The tracts of the wounds were pale and smooth, and only moistened with a serous fluid, and none of the usual appearances of inflammation were visible. A very common occurrence in fishes, is the existence of worms, which perforate the tunics of the alimentary canal, without producing any change of structure, except an increased vascularity around the perforations. The reproductive power of fishes is confined to their fins, which are sometimes regenerated after being lost by accident, or by a species of death which is quite different from that which is the consequence of inflammation in the higher classes of animals.

I have never seen any appearance of inflammation in *reptiles* after wounds or injuries. *Serpents* often lose a portion of their tail; and although there is no attempt made for its reproduction, it is very speedily cicatrized without inflammation. Some *lizzards* are able to reproduce parts that are lost, though not so perfectly as in the lower classes of animals. When lizzards get a new tail, it wants the vertebræ. The *salamander* has more power of reproduction than any other of the class, being capable, according to the statement of many physiologists, of regenerating the tail, the limbs, and the lower jaw. I have seen imperfect attempts at the reproduction of the fingers and toes in the *toad* and *frog*. In all this class of animals, there is great tenacity of life, and power of repairing the effects of injury, though not always the ability of regenerating limbs. Having had occasion to make the experiment of removing a part of the brain of a *toad*, it became necessary to take away a considerable portion of the skull. The wound never seemed to inflame. In a very short time it was healed, and the vacancy in the skull was made up by a substance half cartilage and half bone, leaving, however, a depression, corresponding to the quantity of brain removed." 5.

In birds external mechanical injury produces indubitable inflammation; but the instances in which internal disorders become a cause of inflammation, are very limited, and are nearly confined to febrile states and particular epidemics.

Quadrupeds are subject to inflammation both from external injury and internal disorders; they usually, however, shew but little constitutional sympathy with local disease.

Man is especially prone to inflammation, as well as to constitutional and sympathetic disturbance.

Upon these data and premises, Dr. Macartney founds a much bolder hypothesis than will probably be suspected by our readers. This hypothesis contains the spirit of the book, and embodies the main fact, which its author endeavours to establish. Let him speak for himself.

"The history I have given of the effects of injury in the different classes of animals, proves that the powers of reparation and of reproduction are in proportion to the indisposition or incapacity for inflammation, and leads necessarily to the induction, that inflammation is so far from being necessary to the reparation of parts, that in proportion as it exists, the latter is impeded, retarded, or prevented, and that when inflammation does not exist, the reparative power is equivalent to the original tendency to produce and maintain organic form and structure; that it then becomes a natural function, like the growth of the individual or the reproduction of the species. I am aware that this opinion is opposed to universally received doctrines. The subject, I think, has never been fairly examined. The necessity of some degree of inflammation to the process of reparation has been supposed by the early surgeons, and has been received by the moderns without inquiry. The opinion arose in those rude ages of the art when nothing was trusted to nature, and when the treatment of every wound was such as to induce and maintain for a certain time the most severe inflammation. The ignorance of the ancients of the use of the ligature for suppressing hæmorrhage, led them to employ, instead of it, strong compression, and the actual cautery in cases of wounds and after amputation; and being accustomed to see wounds ultimately heal after such barbarous treatment, they naturally supposed that inflammation and all its evil consequences were necessary; which they took care to ensure in all cases, by boiling oils, hot and irritating ointments, tents, setons, and strict and cumbrous bandages." 7.

After proceeding to notice and partly to criticise the views of Mr. Hunter, and to point out inconsistencies between his doctrines and his facts, Dr. Macartney seizes on a passage in Sir Astley Cooper's lectures, as a fair point on which to try the issue—inflammation or no inflammation in the reparation of injuries.

"The doctrine," he observes, (that inflammation is necessary) "has been avowed by Sir Astley Cooper in the strongest language, in his Lectures on Surgery, published by Mr. Tyrrell. The passage may be taken as expressing the general opinion of the profession in this country on the question. He says: '*Inflammation is a restorative process; no wound can be repaired without it; even the little puncture made by the lancet in bleeding, would inevitably destroy life, if this salutary process did not prevent it.*'

I am well pleased, that the doctrine has thus been so clearly and unequivocally asserted, that no doubt can exist respecting the meaning of the author. It is also fortunate, that Sir Astley has selected the wound made in venesection as his example; as I shall afterwards have occasion to quote the healing of this wound, as one of the strongest and most familiar instances of union being effected, without the slightest inflammation." 9.

We would make a few observations before we proceed any farther.

1. We perceive that confusion is already approaching from the vague sense in which the term inflammation is used by the respective parties. Dr. Macartney does not define what *he* means by inflammation—whether certain phenomena, as swelling, redness, &c.; or certain effects, as adhesion, suppuration, or so on. Mr. Hunter, whose want of education was so unfortunate, sometimes employs "inflammation" in one sense, sometimes in another. Yet this makes all the difference, especially in a dispute.

2. We may, nay we must conceive, that though the pathological state of inflammation be excitable in all animals, its mode will vary in the utmost possible degree. In an animal with a distinct circulating nutritive fluid, inflammation consists essentially in an abnormal afflux of that fluid to a part. How different must the phenomena be, in a creature with red blood or white, with warm blood or cold. If this be so, and reason informs us that it must, how essentially must the nature of inflammation be changed when we arrive at animals, as the polype, in which we can detect no circulating fluid at all. If there be no such fluid, it follows of course that there cannot be any partial or abnormal collections of it. Yet the purposes served by inflammation elsewhere, may be served in such animals by a different process—different in mode, but similar in objects. Animals in this category cannot legitimately be compared with those which possess a true circulation, undoubtedly not with the highest of that class endowed with warm red blood.

To determine the precise points of analogy or difference, a series of experiments would be required. But Dr. Macartney communicates none. The actual effects of mechanical and chemical stimuli—the results of various lesions—the changes, if there be such, of disease, are not stated by our author, and do not appear to have been determined. Yet, in the absence of direct experiment and of positive information, we do not feel ourselves warranted in assenting to the position, that nothing analogous to inflammation occurs in the lowest animals.

3. The statement that reproduction is greatest where inflammation is least or is absent, requires something more than that naked announcement. In the simplest animals the whole body is comparatively homogeneous, and there is no centralization of fluids or of organs. All the molecules, then, must have, loosely speaking, the same powers, and the addition of fresh molecules is comparatively simple. But as animals rise in the scale, centralization obtains, the parts are no longer simple, no longer homogeneous, their life consists in a finely adjusted balance and dependence, and the reproduction of lost parts becomes too great an effort. It is unphilosophical to imply that reproduction is less active in these creatures because inflammation is more so. The cause of both phenomena is to be sought in the centralization of the vital powers and organs. The polype may, for aught that appears to the contrary, have inflammation in its own way, though it cannot have the "dolor, rubor, calor, tumor" of man. In the polype, increased vital energy in the molecules of the part, may constitute the essence, and give rise to undoubted effects of inflammation—in man, such augmented local action would be useless, unless combined with correlative augmented energy in the vital organs.

4. When surgeons of the present day assert and believe that inflammation heals the simplest wound, the best informed imply or understand nothing more than this :—*that*, after the simplest wound, an effusion of plastic material takes place, which forms first a bond of union between the divided surfaces, and afterwards is more or less identified with them in structure; *that*, these changes are attended with a certain afflux of blood to the part; *that*, this appears the simplest mode of what in an increased ratio constitutes the pathological state, and gives rise to the effects of inflammation : and *that*, the steps from this restorative adhesion to the more aggravated phenomena of inflammation are so gradual, that it is impossible to fix any positive limit between the reparative and inflammatory process.

The second Chapter is on the Phenomena of Inflammation. Dr. Macartney commences by digressing to the hypothesis of the cause of animal heat. If we have observed some indications of a tendency to confident generalization in the previous chapter, those indications are rather augmented in the present one. The opinions of physiologists are daily leaning more and more on the connexion between animal heat and respiration—a connexion supported by a broad survey of the animal kingdom. But Dr. Macartney decides in the negative. It would be foreign to our purpose to enter on so important and extensive a question. We must content ourselves with stating that we do not and cannot go along with our author in several of his opinions and conclusions. We may observe that Dr. Macartney is disposed to ascribe the increased heat of inflamed parts, more to their state of local or organic sensibility, than to the condition of their arteries, as regards circulation or secretion—an opinion which is certainly less simple and less intelligible than the commonly received one.

Dr. Macartney treats seriatim of the signs or symptoms of inflammation —heat, pain, redness, swelling, and altered or suspended natural secretions. On each, his remarks are ingenious. We pass to the next Chapter, on the—

### Consequences of Inflammation.

Dr. Macartney very properly observes that, what are denominated the phenomena of inflammation are the *immediate effects* of the augmented sensibility and circulation in the parts engaged. To these succeed, or rather, there arise out of them as a matter of necessity, if the inflammation persist for any time, a number of consequences. These he enumerates as *chemosis*, *œdema*, *vesication*, *suppuration*, and the total *disorganization* and *death* of the part.

1. *Chemosis.*—"When *chemosis*, or the extravasation of the blood, takes place without a rupture of the vessels by mechanic injury, it is not designed to become organized, and therefore it remains in a fluid state. The absorption of the blood, when shed under these circumstances, is tedious. I have known chemosis arising from erysipelas of the face, remain for months before it was removed. When blood is found extravasated in an inflamed part, there are always, I think, ruptured vessels. Such effusions take place under circumstances favourable to the laceration of the small arteries; as when inflammation is very violent, or occurs in lax cellular tissue; or where parts are not yet supported by the deposition of coagulable lymph. Thus, the mucous membrane and the surface of ulcers, when highly inflamed, yield blood. The loose cellular membrane joining the conjunctiva to the globe of the eye, becomes charged with blood in severe ophthalmia, and the submucous tissue of the great intestines is similarly affected in acute dysentery. In the first steps towards the formation of an abscess, before the parts are made solid by coagulable lymph, blood is commonly extravasated.

In some abscesses, as those of the liver, spleen, and brain, we sometimes see with the naked eye the lacerated vessels; and in the first, I have observed the biliary vessels also to be broken, and the bile mixed with the blood and pus contained in the cavity of the abscess. The best evidence is to be obtained by injecting any part, in which blood has been extravasated, when the fluid injection will escape through the broken vessels, and imitate the previous effusion." 24.

It will be observed that Dr. Macartney employs the term Chemosis, to signify extravasation of blood. Ecchymosis is the designation usually given to that pathological condition, and chemosis is commonly understood to mean effusion of serum into the submucous cellular tissue, with inflammatory redness and turgescence of the mucous tissue itself. We mention this, to prevent misconception on the part of our readers. An observation of Dr. Macartney's would imply that he considers chemosis in the eye as purely extravasation of blood. There is some confusion in this.

2. *Œdema.*—This, says the Doctor, is the diffused extravasation of serum. It is usually found in relaxed and depending parts. It may occur with a very low degree of inflammation, as in some species of erysipelas. Persons are most prone to œdema in inflammation, who have the small veins full, or who have a tendency to anasarca; and the parts of the body where œdema is most remarkable, are those, in which the cellular membrane is most lax, as the eye-lids, the prepuce, the scrotum, and the external labia of females. Œdema is always unfavourable to reparation, and parts once affected with it, are apt to retain some serum in the cellular membrane afterwards, giving them a bloated or tumid appearance. Where the disposition to œdema is general in the body, it is a proof of weakness, and often leads to a fatal result, after severe accidents or surgical operations.

3. *Vesication* is familiar. It may result from simply increased irritation in the vessels of the cutis—or from inflammation in constitutions or parts which are incompetent to effect reparation ; so it accompanies mortification.

4. *Suppuration.*—Dr. Macartney considers this as always arising from more or less of inflammatory action. His remarks upon the process need not detain us.

Speaking of *acute abscess,* our author offers the following rationale of the observable phenomena. In the first instance always, he says, some of the small vessels give way, and some blood and serum are poured out into the surrounding tissue. In order to separate the disorganized from the healthy structure, lymph is shed, by which the extravasation of the blood and serum, is restricted within certain limits. This lymph next acquires vascularity and organization, and then, and not before, the secretion of pus commences. In the first stage of abscess, if the fluid be evacuated, it is well known to consist of blood and serum streaked with pus. As the lymph which is designed to compose the walls of the abscess advances in organization, pus of a better quality is secreted; and as the contents of an abscess are proved to be, like the other parts of the body, transitory in their existence, while fresh pus is being added, the original contents are removed by absorption; hence, an abscess of some duration is found only to hold genuine pus. During the time that this change is taking place in the fluid contents, a similar one is proceeding on the solid walls of the abscess. On one side, the process of absorption is reducing the thickness of the parietes, while the other sides are increasing in the same proportion, by the addition of new substance. There is also another remarkable circumstance attending the progression of an abscess, which was first accurately described by Mr. Hunter. On the side of the abscess that is becoming thinner, there is also a disposition to yield, or to be extended; and on the side that is growing, there is a tendency to contract. The pus of an abscess is, therefore, brought to those surfaces on which it is to be evacuated, by four processes—absorption, new growth, extension, and contraction; and as the object to be attained in this case, is the removal of the fluid, the same means are employed for conducting extraneous substances out of the body.

It is not generally supposed that the effusion of lymph and its vascularization are necessary for the secretion of pus. The observation of Dr. Macartney, if well founded, is interesting. Yet, as we see pus formed by mucous membranes, it is not impossible that other tissues when inflamed may do so by their own vessels independently of effused lymph.

*Chronic Abscess.*—When the attempt, continues our author, is made to form an abscess by weak or scrofulous constitutions, and in situations where the cellular substance is lax, the progress of the disease is very different from that above described. The first extravasation is serum, which passes easily into the large cells of the cellular membrane, with little or no injury to their structure; the parietes of the chronic abscess are not composed in the beginning of organized and vascular lymph; no genuine pus therefore is found in such cavities in the first instance; the fluid they contain is serous, mixed with coagulable lymph, parts of which are found as flakes floating in the serum. As the cavities of chronic abscesses are not provoked, either by severe tension, or the quality of the contained fluid, there is no preparation made for some time to remove their contents. These collections therefore often traverse a considerable distance along muscles or under plates of fascia, before they arrive at the skin, which ulcerates very slowly; after which the cavities may inflame, their interior surface become more highly organized, and secrete genuine pus.

He proceeds to remark that sometimes these abscesses, even when of considerable size, are absorbed.

"I have known this to occur several times in psoas abscess, where there was no disease of the vertebræ. In one instance a young lady had one of these languid abscesses formed suddenly above the clavicle; it descended behind the clavicle, and proceeded underneath the mammary gland until it came to the waist, where it was finally removed by absorption; the patient's constitution was strengthened, during this time, which I have found in several instances effectual in causing the absorption of such collections." 34.

Dr. Macartney offers nothing new on the termination of inflammation in mortification, or disorganization of a part.

### ON THE REPUTED CONSEQUENCES OF INFLAMMATION.

Dr. Macartney apologizes for including under the head of reputed consequences of inflammation, the *effusion of coagulable lymph*, and *ulceration*. These, he says, are processes which, from being sometimes associated with inflammation, are ascribed to an inflammatory action, although in their own nature they are perfectly different. This is a startling assertion, particularly when coming from a man like Dr. Macartney. Our readers will be naturally inquisitive to learn his proofs.

"It is well known," he argues, "that coagulable lymph may be thrown out by a natural and healthy action, as in the formation of the decidua uteri: that it is eminently conservative, in arresting hemorrhage from opened vessels; in the union of all the soft parts when divided; in forming the medium of conjunction of fractured bones, and in constructing the walls of an abscess, and of an aneurismal sac. Immediately on the receipt of an injury, also, lymph is shed before there is time for inflammation to set in. The surface of a wound that does not bleed is covered by a layer of lymph, in the very moment that the injury is inflicted. The inflammation which would ensue from the opening of a serous cavity is sometimes altogether averted, and almost always restrained within certain bounds by the effusion of lymph, uniting the opposed surfaces with each other." 38.

After noticing the case of hydrocele, cured by an operation and disposing of it as an objection to his views, Dr. Macartney goes on to remark:—

"It is true, there are some cases of adhesion, which are highly detrimental to the parts concerned. Wherever freedom of motion is necessary to the functions of parts, adhesion may be inconvenient, or fatal. Thus, the iris has its office destroyed by being bound to the adjoining parts; the actions of the heart are embarrassed, by extensive adhesion between it and the pericardium; and I have known the general union of the peritoneal surfaces of the intestines cause strangulation of the whole alimentary canal, and death. It is also the agglutination by lymph, which is the most frequent cause of hernia becoming irreducible, and occasionally of the protruded parts being strangulated. The effusion of lymph in the trachea during croup, causes as much danger as the inflammation: so likewise, when the bladder and urethra are blocked up with lymph." 39.

But, he continues, the circumstance of evil occasionally resulting from adhesion is no proof that adhesion is the consequence of inflammation; and he cites the case of closure of the glottis against irrespirable gases, as analogous—a closure salutary in its intention, though actually endangering life.

Such are the grounds on which Dr. Macartney rests, in excluding the effusion of coagulable lymph from among the products of inflammation. They do not appear to us to be satisfactory.

1. Inflammation is a positive pathological state, marked by certain signs and symptoms. If, after those signs and symptoms, we find pus, or serum, or mortification, we conclude those states to be consequences of inflammation. It signifies nothing what may be the tendency of those consequences, whether salutary or pernicious to the individual. Whichever they may be, they are sequences of the state which we call inflammation.

2. So far as we can see, there are just the same grounds for considering coagulable lymph such a sequence, as for believing serum or pus to be so. A man has a sword run through his belly or his thorax. The familiar signs of inflammation follow, and we find, after death, serum, and lymph, and pus in the peritoneum. Why should we say that the first and the last are the consequences of inflammation, while the second is not? Take rheumatic pericarditis, or acute pleurisy, or even inflammation of the cellular membrane. During life and after death there is every possible evidence of the existence of inflammation, and we find lymph in abundance. These are positive facts, which it appears to us impossible to disprove, and almost equally impossible to explain on any other supposition than the one generally entertained.

3. It is no answer to say that lymph is usually salutary in its operation. That may or may not be the case. When lymph is effused on a mucous membrane or even around it, or in the cranial cavity, it may be pernicious. And pus may be proved by the same line of argument to be no consequence of inflammation; for the formation of pus by an inflamed mucous membrane is infinitely more salutary than that of lymph. Were the latter the usual sequence of inflammation in the urethra, the consequences of a common clap would be formidable.

4. The argument employed by Dr. Macartney, based upon the occasional effusion of lymph as a natural action, is more specious than conclusive. Either, in those instances, the usual phenomena of inflammation precede the effusion of lymph, or they do not. If they do, then the case is one of inflammation—if they do not, then the case is obviously different from that in which they do. Neither in logic nor in reason can it be allowed, that, because in one case inflammation does not precede the effusion of lymph, while in the other it does, therefore in the latter inflammation does not produce the effusion. To make this argument available, it should be shewn that, in both cases, the effusion is similar in degree and kind, and that in the latter the same circumstances exist, to operate as causes, which exist in the former.

5. The truth is, that from the simple cut, producing an effusion of the liquor sanguinis, up to the extensive and violent inflammation of peritonitis, is not a sudden jump, but a series of gradual transitions. The mere interruption of continuity of a few small vessels, and the slight stimulus of a trivial injury, give rise to the effusion of no more lymph than is sufficient to glue the edges of the wound. A more extensive lesion, particularly of certain tissues, leads to positive inflammatory action, and to the effusion of lymph in such an injurious quantity. But, as inflammation, in the first instance, is only an augmented capacity and action of the blood-vessels, it is obvious that there may be any degree of it between their normal standard, and the maximum of which they are capable. We think it would be just as unphilosophical to deny that the effusion of lymph is a common consequence of inflammation, as to assert that inflammation to any amount *must* precede it. It is impossible to maintain the latter position, until we determine with precision the limit between non-inflammatory and inflammatory augmented action. That has not yet been done.

*Ulceration* or *ulcerative absorption* is equally denied, by Dr. Macartney, the right of being a result of inflammation.

The same spirit of argument is employed by our author in this as in the former case, and mutatis mutandis the same mode of reply may be resorted to. The dispute is, perhaps, more verbal than substantial; yet, as we think that Dr. Macartney's views are calculated, in many respects, to breed perplexity, we cannot avoid objecting to them. It would be uncandid not to admit that Dr. Macartney reasons with great ingenuity, that many sound and excellent reflections are mixed up with his hypothetical opinions. All we think it necessary to quote from the remarks on ulceration is the following passage;—

The term *ulcer* is not a correct one. It expresses only a part of the history of the object, to which the name is given; or applies to that state in which the ulcerative process only is going on; perhaps, we might say, that in all instances, where either inflammation or morbid structure does not prevent it, the phenomena that belong to an ulcer, are more reparative than destructive; since in many cases of ulcers tending to cure, there is only that degree of interstitial absorption of the granulations which serves to approximate the edges of the sore, thereby diminishing the magnitude of the cicatrix. An ulcer, therefore, as it is usually presented to our observation, is the result of a compound, or rather opposed action, as the granulative, succeeds the ulcerative processes.

### OF THE DIFFERENT MODES OF REPARATION.

Discarding the classification generally received of union by the first and by the second intention, Dr. Macartney proposes his own.

Re-union, he says, and re-organization are effected in four different ways, which may be designated in the following manner:

*First,* immediate union without any intervening substance such as blood or lymph.

*Second,* the union by the medium of coagulable lymph, or a clot of blood.

*Third,* re-organization without any medium of lymph or granulations, the cavity of the wound becoming obliterated by a natural process of growth.

*Fourth,* the reparation by means of a new, vascular, and organized substance, called granulations.

Speaking of the organization of effused lymph, Dr. Macartney makes the following observations, with which we are disposed to agree, in spite of the opinions of Müller to the contrary.

" Mr. Hunter assumed, that the vessels arose in the lymph, and subsequently established their connexion with the vessels of the part, because, he observed, that vessels began to form in the membrane of the incubated egg, before they existed in the fœtal chick. There is a great difference, however, between the original formation of vessels, and the acquisition of vascularity by lymph, deposited in contact with surfaces that are already organized; and it is more difficult to imagine, that vessels should commence in a clot of lymph or blood, than that they should be extended into it from the adjoining surfaces. It is also impossible to conceive that the thin layers of lymph which unite serous membrane, or the effusion which consolidates cellular structure, do not obtain their vascularity from the adjoining parts. Further, I have seen vessels passing for a short way into a clot of blood, covering the surface of an ulcer, when the coagulum possessed no vascularity of its own. I have also succeeded in forcing injection into the coagula formed in the cavities of the heart after death, which injection presented the appearance of red elongated lines." 51.

Reparation by the modelling process, has never, says our author, been described. However, when healthy parts are injured, although it may be to the greatest extent, if placed under the most favourable circumstances for carrying on their natural actions, the process of reparation is nearly the same as in animals of a simple structure.

"The pain arising from the injury soon ceases. No tumefaction ensues, separating the edges of the wound, and its surfaces are not only disposed to lie in contact, but even to approach each other so much, that they cannot be kept asunder by mechanic restraint; there is, therefore, no necessity for the effusion of lymph; and as there is no cavity to be filled up, granulations are not formed. The surfaces of the wound, although they come into contact, do not unite by vessels shooting across; they are smooth, red, and moistened with a fluid, which is probably serum, and present the appearance of one of the natural mucous surfaces of the body. If any parts have been killed by the injury, they are separated, by simply as much interstitial absorption as is sufficient to set them free. The wound is finally healed by the same means which determine the shape of the natural parts of the body. It gradually diminishes in extent until it is obliterated; or it may be cicatrized before the surfaces are abolished, after which the same process of natural growth goes on, until no part of the original wound is left. The cicatrix which succeeds the cure of injury by the modelling or growing process, is small, pliant, free from those callous adhesions to the parts underneath, and the morbid sensations that so often belong to those cicatrices, which have for their bases the deposits of lymph, or the new formed structures called granulations. When the modelling process or cure by natural growth goes on perfectly, there is no inflammation in the part, and the patients are so entirely free from all uneasy sensations, that I have known instances of their being ignorant of the real site and extent of the injury, until they had examined the part with their hand, or saw it in a looking-glass." 54.

We confess that we are not familiar with this mode of reparation, and we cannot therefore offer an opinion on the fidelity, or otherwise, of Dr. Macartney's description of it.

Dr. Macartney's observations on granulation are ingenious, but do not require particular notice from us.

Passing over the chapter on Cicatrization, we pause at that on—

### Reparation in different Tissues.

Different tissues possess different powers of reparation. It is difficult to refer those differences to a general law, or to express the facts by any simple formula. But the simpler the tissue, the more perfect is its reparation. It is both curious and instructive to examine the reparative process in each tissue.

A. *Nervous Tissue.*—Vacancies in the *brain* are not filled up by any substance, bearing a resemblance to the cerebral tissue. The *spinal marrow* also is never regenerated.

All wounds of nerves heal by the formation of a hard bulb or nodule; this exceedingly firm and condensed structure is found uniting the two ends of a divided nerve, and also surrounding the part of it which has been cut in amputation; therefore, this is the natural mode of the healing of a nerve, whether by the medium of lymph or granulations. Though visible nervous filaments have been fruitlessly sought for in the nodule, sensation and voluntary motion are restored, sooner or later, beyond it. In some parts, the nerves naturally present a very condensed structure—the posterior tibial nerve, for example, below the inner ankle.

"When the nerves are divided some way from the end of the stump, I have been able to trace the appearance of filaments radiating from the bulbs, and proceeding to the skin. A preparation of this kind, in the stump of a finger, was preserved in my anatomical collection, which is now in possession of the University of Cambridge. Professor Muller states, that his assistant, Dr. Schann, was able to dissect filaments passing from one end of a divided nerve to the other in the frog. Also, it cannot be doubted that the sensibility of granulations is caused by the extension of nervous filaments into them.

I once had the digital nerve of my thumb divided by a wound. The sensation was not perfectly recovered for some years, but is now as complete as before the accident. I observed a curious fact on this occasion. I was aware, from the depth of the wound, that the nerve must have been divided, and I was surprized that the sensation was not instantly lost; but, while examining the part, the sense of feeling vanished, giving me the impression of a blast of air having passed from the thumb up my arm. This occurred in about two minutes after receiving the wound." 68.

B. *Muscular Tissue.*—Probably this is never perfectly restored in the higher classes of animals. The deficiency is commonly seen to be partially filled up by a pale substance, which appears to be coagulable lymph imperfectly organized.

C. *Fibrous Tissue.*—When *tendon* is divided, and afterwards united, it is by means of a newly-formed condensed substance, not possessing the true structure nor the brilliant metallic lustre of tendon, yet sufficiently strong to perform its offices. If tendinous structure do not unite, as in the laceration which occurs in a dislocation, the ends of the fibrous tissue form a number of tags or little bulbs, by the effusion of coagulable lymph; these become very hard, and finally smooth on the surface. It is said that the *dura mater* is not reproduced. A dense membrane, however, is formed, which answers a similar purpose. The only fibrous structure which is perfectly regenerated, is the *periosteum.*

D. *Cartilaginous Tissue.*—The *cartilages* covering the ends of bones, when destroyed, never appear to form again. In old persons, especially, the place of the cartilages of the joints is often supplied by the conversion of the osseous tissue into a substance possessing the density, hardness, and polished surface of china or white glass. Yet, in *false joints,* the ends of the bones that rub on each other become covered with a sort of spurious cartilage.

A vacancy in the cartilages of the larynx is filled up by a tough dense cellular substance. The cartilaginous portions of the ribs are not reproduced by genuine cartilage, and the union of these parts is often confirmed by being surrounded with a shell of bone.

Dr. Macartney doubts whether the place of the fibro-cartilages is ever supplied, except by a tough cellular structure, not unlike common ligament, but wanting its brilliancy.

E. *Serous Tissue.*—This seems as perfectly restored as any tissue, saving, perhaps, the cellular.

F. *Cutaneous Tissue.*—Neither the skin nor mucous membrane appears to be perfectly restored. The villous surface is not regenerated.

G. *Arterial Tissue.*—The middle coat has not been observed to be regenerated. The puncture of an artery is closed by condensed cellular membrane.

H. *Venous Tissue.*—"The proper tissue of the *veins* never grows again. I have always observed that veins on which venesection had been performed, presented on the inner surface a depressed line corresponding to the incision made with the lancet. The vacancy in veins being always closed by the cellular membrane somewhat condensed, accounts for those little pouches that are occasionally perceived over the vein, when the edges of the puncture have not been brought together after the operation of bleeding. These pouches are often seen, and sometimes of a large size, on the veins of horses, in consequence of the mode employed by farriers of pinning the edges of the wound, made in bleeding these animals, and the horse being allowed to stoop the head after the operation. There is no true venous tissue found in these pouches." 71.

I. *Osseous Tissue* is repaired by bone, in a manner that we need not specify. We regret that we have not space for Dr. Macartney's observations on necrosis. At a future opportunity we shall advert to them.

K. *Epidermoid Tissues.*—The reproduction of these, subjected to attrition, &c. as they are, is usually a process similar to that of their growth. But when the secreting apparatus is destroyed, the product cannot well be reproduced. Thus, when the capsules which contain the roots of the hairs are destroyed, the part continues bald.

"If the vascular surface which furnishes the horny substance of the nail, be completely destroyed, no new nail is usually formed; nevertheless, there appears to be sometimes a great disposition to the revival of the proper structure for secreting the nail. It reappears, in some cases, after caustic has been repeatedly applied to it, to prevent the reproduction of a nail which penetrates the skin. Two instances are recorded of a nail growing on the stump of an amputated finger; and I had a preparation of a finger, which had the last and a part of the second joint removed by amputation. The proper vascular structure had been produced on the stump, and a true horny nail generated; which, however, had a deformed appearance, and was very much hooked. This preparation is now with my collection placed in the University of Cambridge." 77.

As nail is only a modification of the horny or epidermoid tissue, we cannot be astonished at the occasional production of a modification of it. We say modification of it, for it is unlikely that perfect nail could be formed without a matrix. Dr. Macartney admits, that, in his case, the nail had a deformed appearance, and was very much hooked.

We arrive at the Chapter on the—

### CONSTITUTIONAL OR REMOTE CAUSES OF INFLAMMATION.

This contains some ingenious hints mixed with much that must necessarily be familiar. We shall only pick out one or two insulated passages for notice.

*a.* Speaking of sympathetic morbid sensations, Dr. Macartney remarks :—

"I remember a very curious instance, in which I discovered a stricture in the œsophagus, by the person feeling an acute pain in the little finger of the left hand every time he swallowed." 84.

*b.* It is well known that the suppression of natural secretions or of habitual discharges, may prove a cause of inflammation. The explanation generally consists in supposing that the blood or the system becomes charged with the peccant matter previously evacuated. But Dr. Macartney observes, that, generally speaking, the mischief occurs before there is time for the circulating fluid to become redundant, in consequence of even extensive secretions being interrupted; and in many cases, the discharge which is stopped, is so insignificant, as to quantity, as to be incapable of producing any influence on the mass of blood.

We must, therefore, he continues, adopt some other explanation for the suppression of secretion becoming the cause of inflammation, than the disturbance of the balance between the quantity of the circulating fluid, and that of the discharge which has been suspended. It is probable, that the interruption of the secreting function, in any one part of the arterial system, and especially on sentient surfaces, may, from the law of sympathy, which is so remarkable between the vascular system and these surfaces, become a direct motive to the arteries to assume, in certain places, an inflammatory state. This mode of explanation is rendered more probable, from the fact, that the removing very slight external irritation, or inflammations, which furnish very little or no pus, sometimes causes the most severe constitutional disturbance, which is removed by the recurrence of the local irritation.

We think there can be little doubt of the correctness of this explanation. It is not the mere suppression of discharge, but the alteration or repulsion of a mode of action that affects the system. Suppose some habitual vascular excitement or congestion in a given point. If that is suddenly repressed, some other part in the vascular circle, by the operation of the perplexing law of sympathy, becomes the seat of congestion or excitement. That vascular plethora is mixed up with this, seems however, to be proved, by the security which, under such circumstances, evacuant medicines offer.

"I have seen," says Dr. Macartney, "the most serious diseases endangering life, induced by the cure of that slight cutaneous affection of the cheek, which sometimes is a mere efflorescence, periodically throwing off a little furfur, or forming a crust. Dr. Jenner once shewed me a red spot on his wrist, that could be covered with a sixpence; he said, whenever it faded, his stomach and general feeling of health were affected." 87.

But in cases like Dr. Jenner's, it is more likely that the general disturbance was due to some modification of the state of the internal organs, than to the influence of so trivial a cutaneous eruption. The latter was in all probability a symptom. When present, it shewed a condition of internal organs compatible with health. When a contrary condition of those organs ensued it subsided.

c. Dr. Macartney is eloquent on the subject of "taking cold." Of all parts of the body, he says, the back is the most susceptible of cold, thus insidiously applied, and the chilliness is sometimes even felt first along the back, although it may not have been the part immediatèly exposed. Perhaps this circumstance depends on the nerves of the skin of the back having so direct a communication with the spinal-marrow.

Dr. Macartney follows this up.

"In the same manner, when a person is placed in the situation favourable to giving cold, the danger may be avoided, by turning the face instead of the back to the direction of the stream of cold and damp air, observing the sensation caused by the impression, and voluntarily rousing himself to a feeling of resistance. In protecting the body by clothing, the back is the part chiefly to be regarded, and hence winter waistcoats should have the back made of at least as thick materials as the front. People commonly fall into the error of clothing the front of the body warmly, and expect to escape cold by muffling the throat. The Irish peasantry more wisely, expose the throat and breast, and defend the back by a warm great coat, which they seldom close in front." 89.

So that the idea of Pat's having "his coat buttoned behind to keep his belly warm" is a libel on him. He keeps his "belly warm," by not buttoning his coat at all.

The Local Causes of Inflammation form the subject of the next Chapter.

Dr. Macartney's observations are both numerous and judicious. But they are scarcely suited for our pages, containing, necessarily, much familiar matter. We may quote the following passage, with the expression of a hope rather than a confident expectation, that Dr. Macartney's ideas may not be of too sanguine a complexion. Speaking of the effects of dead animal matter introduced into the body or merely applied to the skin, he observes:—

"Every anatomical class has annually furnished examples of the effects of this species of infection, except the dissecting class of the Dublin University, during the time it was placed under my direction. Very simple means of prevention were employed, with so much success, that no severe disease from wounds received in dissection occurred, (when the proper means of prevention had been employed,) during the last fifteen years that I held the Professorship of Anatomy in that University. The means resorted to consisted in immediately washing the wounded part, and afterwards keeping it wet for a few hours, with a solution of alum in water. Most probably, any other fluid, such as the liquor plumbi, solution of sulphate of zinc, &c., which would have the effect of coagulating the animal substance, would answer quite as well; but the solution of alum was always at hand, and, I believe, if immediately applied, would never fail in preventing infection." 104.

Dr. Macartney remarks very justly, that the two stages of decomposition in the dead body, which render the animal substance most dangerous, are, that which takes place immediately after death, and the extreme degree of putrefaction. The latter is most apprehended, but, it usually occasions only a diffused inflammation in the part wounded, without any serious sympathy of the constitution.

Some dead animal substances, he goes on to say, are more likely to communicate this dangerous disease, than others. The brain, in the recently dead body, is extremely apt to produce it, even when no wound is received. The sero-purulent fluid, found in the large cavities after death, (if no means of prevention be employed,) seldom fails to infect persons; and the most dangerous animal fluid is that contained in the cavity of the abdomen, after puerperal peritonitis, or the serum found in parts which have suffered diffused or gangrenous inflammation. The white cancer of the liver, and the substance of medullary tumors, are found to be very irritating, when merely applied to the hands, without a breach of surface. Dr. Macartney has several times had his hands inflamed from handling this morbid structure, even after it had for some time been preserved in spirit.

"Some persons are more susceptible of the infection from dead animal matter than others. I have heard of a student who never escaped it, after receiving a wound in dissection. I have, myself, so strong a tendency to be affected by the irritation of animal matter, that I formerly suffered more or less every year from this cause. Since, however, I adopted the means of prevention already mentioned, I never have experienced either local inflammation or constitutional illness, after wounds received in dissection. That there is no diminution of my susceptibility, is proved, by my still having red patches on my hands, which itch and smart, if I dissect a brain, without continually wetting my hands with the alum water." 107.

We have only twice suffered severely from handling dead bodies. It is singular that each time it was after examining a person who had died from scarlet fever, and in neither instance was there the slightest evidence of our having received a wound. On the second occasion the local symptoms were succeeded by a sort of erythematous affection of the throat, a true secondary symptom.

Dr. Macartney remarks that the secretions of the human body, when they are accumulated in foul clothes, occasionally produce a dangerous and obstinate inflammation of the hands of washerwomen. He has never seen this followed by more than common sympathetic fever. But ill-conditioned abscesses may occur, and the limb may even be lost. We have twice seen, in washerwomen, and after washing suspected linen, inflammation of the deep cellular membrane of the hand, diffusing itself along the fore-arm, beneath the annular ligament, and between the muscles. Each case proved fatal.

Dr. Macartney goes on to observe that:—The dead substance of other animals, besides that of the human subject, although less dangerous, is capable of infecting somewhat in the same way. Persons who clean tripes are liable to a peculiar erysipelatous inflammation, which passes up one finger and down another. The same has occurred from paunching a hare, and horse-killers occasionally suffer severe diffused inflammation. The fluids of the recently killed animal are, however, much less irritating, than the same in an advanced state of putrefaction, and hence butchers seldom are known to suffer from wounds received in the practice of their business; although, as already observed, the greatest power of infecting, belongs to animal substance immediately after death with respect to the human subject.

Dr. Macartney does not mention, at least in this place, any special treatment for this affection. Sir B. Brodie recommends the oxymuriate of mercury. We have, on several occasions, seen small doses of blue-pill with saline aperients, and the application of a strong goulard poultice, remove the affection in a day or two.

We proceed to the

### Proximate Cause of Inflammation.

We must pass over the Chapter on this subject. There is much in it, which, if we discussed, we must dispute; and there is also much that is certainly ingenious and probably true. We must pass, too, over the succeeding Chapter on the Species of Inflammation, which contains nothing of consequence.

### On Congestion as contra-distinguished from Inflammation.

It is obviously of great importance to distinguish, during life or after death, the two pathological states of congestion and inflammation. Dr. Macartney devotes a short Chapter to the subject, and points out some criteria which he deems decisive.

Congestion, he rightly observes, belongs to the venous system. It is caused by any mechanical impediment to the free motion of the blood in the veins: such as obstruction to the circulation of the blood in the liver, the lungs, or other important organs, or by pressure on the trunk of any vein. It is also brought on by the suppression or diminution of natural secretions, and by supplying the body with more nutriment than is expended in growth or secretion. It is sometimes induced, and always favoured by dejection of mind and sedentary habits, which serve to accumulate the blood in the venous system, and to embarrass the circulation.

Every impediment to the passage of the blood through the small veins, necessarily renders its passage slow. But the blood appears to be also very fluid, at least it transudes in many cases through the coats of the veins into the neighbouring cellular membrane. The blood, in congestion, is either purple, or still darker; in melæna, the blood poured out is black.

"The most remarkable circumstance, with respect to congestion, and the one which has not hitherto been described, is, that arteries found in a congested part are smaller than their natural size." 140.

After a few other observations, Dr. Macartney goes on to remark:—

"In order to ascertain the direct effect of venous congestion, on the arteries of the part concerned, I made the following experiment: I put ligatures on both the jugular veins of the rabbit; the animal died apoplectic, and upon examining the state of the vessels of the ears, I found the veins, which lie towards the outer edge of the ear, greatly enlarged, and gorged with dark blood; but the artery, which runs in the centre of the ear, was reduced very much below its natural size, so that it appeared as a mere line. The result of this experiment made me wish to see what would be the instantaneous effect of arresting the venous circulation. I accordingly exposed the mesentery in a young rabbit, and having tied the trunks of several mesenteric veins, their corresponding arteries contracted immediately, in the most palpable manner, and to a very small size; as if taught by their organic instinct, that blood should not be permitted to go where it must immediately return.

From the description just given, congestion never should be confounded with inflammation. They are essentially different in all respects: the uniform and dark red colour of a part in a state of congestion, as contrasted with the brighter red colour, and distinctly ramified arteries in inflammation, ought at once be sufficient to point out the difference between these two affections." 141.

Dr. Macartney concludes by laying down the distinction between congestion and determination of blood.

When blood, he says, is sent in too great quantity to any part, it is because the arteries of that part dilate beyond their natural state, in consequence of some excitement of particular organs; and therefore the sensibility, temperature, and bright red colour of the parts are increased: whereas in congestion neither the sensibility nor temperature is augmented. Determination of blood may itself be easily distinguished from inflammation, by the general appearance, by the causes, by the absence of any of the consequences of real inflammation, and especially by the want of the peculiar pain, which would belong to the tissue concerned, if its turgescence or fulness of blood had arisen from inflammatory action.

It appears to us, that determination of blood to an organ, is a state by no means accurately defined, or definable. If Dr. Macartney's account be correct, it is difficult to say in what determination of blood differs from the earlier stage of inflammation. Probably determination of blood is a state which may end either in inflammation or congestion, as concurrent circumstances may determine. The phenomena of several cerebral affections appear to point out a much closer connexion between determination of blood and congestion than Dr. Macartney is willing to allow. We proceed to the last Chapter of the work, which treats:—

### Of the Remedies for Inflammation.

Dr. Macartney classifies these remedies under the following heads:—

1st. Remedies which diminish the force of the heart, and give the disposition generally, to the small arteries to go into the contracted state.

2nd. Means that effect a diminished size of the arteries, or reduce the sensibility in the inflamed part.

3rd. Medicines that augment or reproduce the natural secretions, and thereby abate the circulation, or lessen the effusions made into inflamed parts.

4th. Counter-irritations, secretions, or impressions made in different parts from those which are inflamed.

5th. Lotions or fluids which exert sedative and astringent power.

6th. Means for affecting in an agreeable manner, the sensations of inflamed parts.

7th. Causes which produce an easy or satisfied state of feeling, on the sentient surfaces, or in the individual.

Speaking of tartar emetic, Dr. Macartney professes himself unable to understand on what grounds, the very large doses of the medicine, now so fashionable, are prescribed. Small and frequent doses, he contends, are sufficient to produce all the effects that are desirable. If one grain of tartar emetic be dissolved in a pint of water, and a table-spoonful of this solution be administered every half-hour or hour, an extreme degree of nausea will certainly be excited, with a reduction in the strength and frequency of the pulse, and usually some perspiration. The good effects of nausea depend on its being kept steadily up for some time. But, as Dr. Macartney observes, preparations of antimony act on some peculiar constitutions as a mineral poison, producing an alarming degree of prostration and distress. He has known these medicines also, when long continued, sometimes induce tenderness of the gums, an increased flow of saliva, and a cadaverous fætor of the breath.

*Cold Applications.*—Dr. Macartney makes some good remarks upon these. The sudden application of cold induces a re-action. But it must be remembered that it is the sudden application of cold, *speedily withdrawn*, which does so. If the cold be maintained, there is no re-action. Dr. Macartney advises that the cold should never be suddenly applied, nor suddenly withdrawn. The inflamed part should be as it were seduced to surrender its temperature willingly, until the power of resistance or of reaction be past, and in carrying it back to the natural state, the same principle should be followed.

"The best example of the power of low temperature to cause contraction of the arteries, is seen in what is called *a dead finger*, in which, there is neither circulation nor feeling, a fact I had the opportunity of proving lately by a direct experiment on a person whose thumb was benumbed in this manner. On making a cut into it with a pair of scissors, no pain was felt, nor did any blood issue from the wound, until the sensibility and circulation returned: now, this extreme case never occurs in the most intense frost, but always when the atmosphere is damp, and not severely cold." 157.

We were not aware of the last-mentioned fact, nor does it seem consistent with what we hear of the effects of very low temperature. Our author believes that the remedial operation of a moderate degree of cold is in the majority of cases preferable. It is usually imperfectly attained by the evaporating lotions in common use. Dr. Macartney describes his ingenious method of obtaining, for the part, the uninterrupted operation of cold and moisture.

"The most easy and manageable way of employing irrigation, is to place the limb of the patient in a trough, and having laid some lint on the inflamed part, to let the water be conducted by means of a stripe of woollen cloth, from a vessel holding the water or other fluid, which may be placed on a chair or table standing beside the bed. One end of the stripe is to be inserted into this vessel: the other, which should be cut into a pointed shape, laid on the lint. The water will then proceed in the manner of a syphon continually from the vessel, not by drops falling from a height, the sensation of which is disagreeable. The water is carried off by a tube proceeding from the end of the trough, into a vessel, placed at the end of the bed. I have found that a stripe of cloth of some breadth, where it is inserted into the water and ending in a point, where it touches the lint, answers the purpose of a syphon much better than the filaments of candle wick, which some surgeons have employed. The patient with this apparatus is able to vary his position, which is a great comfort to him. It is obvious, that irrigation can only be used with convenience to the extremities. The water may have any degree of temperature that is desired, and if it should be wished to employ iced water, the vessel holding it may be placed at a distance from the patient's bed, or even outside the room, and conveyed by an elastic tube, on which there is a cock to regulate its admission into a smaller vessel, situate near to the bed." 159.

Our author goes on to state, that the mode of maintaining a continual accession or renewal of a fluid application, may be converted to many useful purposes. Professor Wiedleck has availed himself of it in the construction of his chair: in the back of which is placed a reservoir of fluid, from whence a tube passes underneath the seat, to the front of the chair, where it is connected by a stop-cock with a catheter, which has been previously introduced into the bladder. This catheter is double internally, or has two passages, each with an opening into the bladder, and the handle of the instrument exhibits the two distinct tubes, into one of which, the end of the elastic tube which is furnished with a cock is introduced. The fluid from the reservoir is thus conveyed by one side of the catheter, and is returned by the other, and consequently there is a sort of stream carried through the bladder. He has used the apparatus for chronic inflammation and catarrh of the bladder. And Dr. Macartney has contrived a tube on the same plan, for transmitting a continued stream of fluid through the vagina or the rectum. Dr. M. has likewise invented a glass vessel, something like a cupping-glass, but larger, with two tubes entering it, one which admits the fluid near the top, and another near the bottom that allows it to escape. This vessel was designed for cleansing foul ulcers or cancers; but might be used for the purpose of abstracting the heat, by a stream of cold water, or for administering medicated fluids to external surfaces, if flat, in the same manner as the double canula conveys them to the internal cavities. And he has further proposed, that the principle of the double passage should be extended to the tube of the stomach-pump; by which, he thinks, the effects of the instrument would be rendered more certain and speedy.

Dr. Macartney particularly insists on not applying cold too suddenly, when we wish to get the lowering effects of cold. To obviate re-action, the temperature of the part should be gradually reduced. In all cases where hemorrhage is to be apprehended, the use of ice or iced water is most valuable, and may save the patient from the application of ligatures to small arteries. Dr. M. has treated cynanche tonsillaris by the frequent use of a gargle of iced water, with remarkable success. The disease has been by this means arrested in a few hours. He has also found ice effectual in stopping obstinate hiccup, when all other remedies had failed.

"Ice or iced water is better for the purpose of generating a great degree of cold, than any of the frigorific mixtures. Some of the latter would congeal the part, none of them would be proper with a raw surface, and it is rarely necessary to reduce the temperature in an extreme degree, without the existence of a wound. When they are proper it may be useful to know, that the mixture of five parts of muriate of ammonia, five parts of nitre, and sixteen parts of water, sinks the thermometer from 50° to 10°; equal parts of nitrate of ammonia and water reduce the temperature from 50° to 4°, and five parts of sulphate of soda, with four parts of diluted sulphuric acid, bring down the instrument from 50° to 3°." 161.

Moderate cold is generally suitable when the inflammation is not very violent, and is accompanied with heat. Irrigation with cold water, especially in the Summer months or in warm climates, is the most convenient mode of applying it. As a general rule, the feelings of the patient are a valuable test of the propriety of warm or cold applications.

Waiving the discussion of the effects of mercury, saline purgatives, and baths, we may pause and examine the operation of counter-irritants. We do so, because we are not quite certain that remedies of this description are always employed in a really scientific manner.

Counter-irritants are divisible into mere rubefacients—vesicants, or such as give rise to secretion of serum or lymph, and sero-purulent fluid—applications which induce suppuration and ulceration, as tartar emetic—and applications which first destroy the skin and cellular membrane and give rise to suppuration afterwards, as the caustic potass, &c.

Dr. Macartney observes, that the milder counter-irritations are suited to diseases near to the surface, or which are situated opposite to the skin to which the application is made. The cautery or severe caustics are only justifiable, where the disease to be counteracted has a deep situation, and has a variety of interjacent structures, as in the hip disease and caries of the vertebræ.

He remarks that all counter-inflammations should be of a kind that naturally tend to subside. This is quite obvious. If any artificial inflammation, he adds, be carried so far, as to join or communicate with the original disease, it becomes mischievous; for, it is essential to the operation of any counter-irritation, that the parts placed between it and the internal disease, should be entirely free from inflammation, although they may be very thin, and consequently the two inflammations very near each other.

"If the inflammation from the previous disease, and that induced by the surgeon, be kept perfectly separate, although near each other, the influence of the latter is very considerable in diminishing the former: nevertheless, the original disease does not appear to possess any power over the counter-inflammation, either in diminishing or increasing it. This fact is difficult to explain; indeed, the mode of operation of any counter-irritation is very obscure: perhaps it is because the artificial disease being always disposed to cease or recover, creates the same tendency in the original inflammation." 166.

It does not appear to us that the difficulty is, theoretically, so great as Dr. Macartney represents it. Counter-irritation is principally used and useful for diseases characterised by increased vascular action and determination of blood. If we can set up an action and determination in contiguous vessels, it is easy to suppose that we may diminish the quantity of blood in those concerned in the disease, and consequently the diseased action itself. We see these variations in the balance of the circulation continually. A woman who is menstruating has generally a pallid face and a mottled complexion from the diminution of the vascular supply to the surface. The feet are cold when the cerebrum is oppressed with blood. The same state of the cerebrum is relieved by active purgatives, which determine the blood to the lower bowels. We might multiply instances of this sort, and follow out the argument more in detail; but we think we have said enough to render it probable that the action of counter-irritants is tolerably explicable on admitted principles.

"*Sinapisms* are counter-irritants of more value I believe than is generally supposed; the rapidity of their action is of importance, and the redness which remains on the skin for a considerable time, is an advantage, that does not belong to any of the milder kinds of counter-inflammations. From observing the good effects of blisters in gonorrhœa, gleet, and irritable bladder, I am disposed to think that sinapisms might be more useful in those diseases. I have found sinapisms very effectual in removing the inflammation of the tonsils in some cases." 167.

Of blisters, Dr. Macartney says that, in general, they are suffered to remain too long before they are cut, unless it be in persons with strong skin.

It is usually sufficient that the skin be inflamed, the serum will continue to flow at the first and second dressings. When it is designed to heal a blister soon, the water dressing should be used in place of any ointment. With some persons, he continues, blisters continue to form serum, and there is no tendency in them to heal. In one instance the blister remained open for weeks. In such cases the discharge is speedily stopped, and the blister healed, by sprinkling over its surface, a powder composed of equal parts of lapis calaminaris and cinchona, and using the water dressing over the part, to prevent the powder encrusting on it.

Dr. Macartney states that there is a plaster made by only one person in Paris, for maintaining a purulent discharge on a blistered surface, which is much preferable to savine cerate. It is paper on which there is a soft plaster very thinly spread; there are two kinds, one milder in its operation than the other, but they both keep up a secretion of pus, from a raw surface, apparently for any length of time, without inducing as much irritation as the savine cerate occasions in a few days. It is made and sold by *Mr. Albespeyres*, Rue du Faubourg St. Dennis No. 84, Paris. The composition of the plaster is a secret.

Such are the principal remarks which Dr. Macartney offers upon counter-irritants. We would observe that à priori it appears reasonable, and experience, we think, bears out the presumption, *that* the mode of counter-irritation should have a sort of physiological relation to the primitive morbid action. Thus, in diseases characterized by a tendency to effusion of serum and lymph, blisters are advantageous; inflammations of the serous and synovial membranes are examples of the fact. In chronic diseases, especially in such as are disposed to end in the formation of pus, those counter-irritants which produce a secretion of pus from the surface generally answer best; thus in ulceration of the cartilages of the joints and in caries of the vertebræ, setons or issues are preferable to blisters. In the slighter morbid actions which consist of determinations of blood, rubefacients are often sufficient. In the more sudden or severe determinations, which amount to inflammation, the positive abstraction of blood from the neighbourhood or the large vascular trunks is indispensably requisite. Without pushing this view of the subject too far, we are justified in recommending it as a general principle of action, and in considering it far from useless.

Dr. Macartney proceeds to the *Different Medicated Lotions.*

He speaks very highly, and we cordially agree with him, of the dilute liquor plumbi subacetatis. The following, however, is a greater amount of success than we should have expected from it.

"The lead lotion never fails to cure tinea capitis, however long and obstinately the complaint might have resisted other remedies, provided the application of the lotion be properly conducted. The hair should first be cut close to the head, but need not be shaved off; water dressing or a poultice of any kind is then to be applied, merely for the purpose of cleansing the skin of the crusts, and all other impurities. There will then be seen under each crust, a red spot of the skin, denuded of its cuticle, and the villous surface exposed. The lotion should now be applied by means of lint thoroughly wetted with the fluid, and covered with a plate of Indian rubber, or a piece of oiled silk to prevent evaporation. Every time this dressing is changed, which should be very frequently at first, the head should be washed with some of the lotion, and the lint should be replaced by some that is clean, which is to be completely wetted with the lotion, and covered as before." 173.

But the application must not be suspended for one night, nor even for a few hours; the crust will re-appear if it is so. We have often used the lead poultice for eczema of the scalp, but we cannot ourselves speak positively to its utility in genuine porrigo.

The next set of local remedies to which Dr. Macartney proceeds, is that which gives rise to *an agreeable state of feeling* in a part or in the whole of the body. The most powerful of these means is *steam;* Dr. Macartney describes a simple mode of applying it, for which we must refer to the work itself. The use of *water* at all temperatures, is strongly advocated by our author, and the *water dressing*, already often alluded to, is his favourite method of employing it.

"The substance that I have generally made the immediate object of application, is the finest and softest lint; and for the covering material, either oiled silk, or a thin plate of Indian rubber. Simple as this mode of dressing may appear, it requires to be managed with care, and attention to many circumstances, which would appear trivial, to persons unacquainted with the nature of the remedy. Two, three, or four layers of the lint should be first folded together, according to the size of the part to be covered, taking care also that the soft side of the lint is the outer one. In wetting the lint the first time, it is necessary to either float it in the water, before folding it, or if it be first folded, it should be pressed between the fingers, to urge the fluid into the interstices of the lint, which receive fluid with difficulty, until all the air they contain be expelled. The lint, when applied, should just contain as much water as not to drop. The oiled silk, or Indian rubber, should project so much beyond the margin of the lint, as may prevent evaporation, which will vary according to the shape of the part on which the dressing is laid, and, the thickness of the folded lint." 181.

The lint should be wetted, without any bandage, as that might rise to a feeling of constraint. It is, therefore, sometimes necessary to stitch the silk into a particular shape. As a general rule, the lint should be changed three times during the day and twice during the night. In cases, where the inflammation is moderate, and the skin unbroken, the dressing will only require to be changed every twelve hours. At each time that the dressing is renewed, the lint and oiled silk should be carefully washed, and when it is applied to ulcers, fresh lint should replace that taken off, the utmost cleanliness being of the first importance. French oiled silk is much superior to English, as it does not adhere to the skin. When it is desirable to combine cold with the water dressing, a bladder holding iced water may be laid over the oiled silk, or where the comfort of warmth may be required, the dressing may be covered with flannel.

Dr. Macartney denounces poultices. It seems to us that he does them an injustice, when he declares that a poultice is made of materials, which, in a term far short of its renewal, become sour, and thereby render the poultice, after the first few hours, an irritating application. The greasy substances which are added to prevent the poultice adhering to the skin, do not always answer the end, and soon become rancid. A poultice favours the formation of pus, and causes a throbbing or pulsating pain, and a feeling of tenderness in the part, which are the natural attendants on the process of suppuration. It imbibes the pus it serves to create, and thereby becomes more irritating.

A poultice, before it is many hours on, is a mixture of sour farinaceous substance, rancid oil, and pus, oppressing the part by its weight, and beginning to adhere round its edges to the skin, creating a sense of constriction. In proportion to his dislike of poultices is his regard for water dressing. He speaks in the most laudatory terms of it. That he is well disposed to believe every thing in favour of it must be evident when he states *that* it puts *boils* completely under our control; *that* he has received numerous accounts of *gonorrhœa* being cured in one or two weeks by the external application of water to the penis; *that* it never fails to eradicate corns, *if* used long enough; *that* ganglia are removed, and loose cartilages, he thinks, may be so too, by it; *that* tetanus can hardly come on when it is employed. All these are confident opinions, and evince a very strong predilection for water dressing.

Dr. Macartney offers some very good observations on the effect of repose, proper position, change of air, exhilaration of mind, confident anticipations, and so forth. The following extract is curious.

"A new, and at first sight, a very singular mode of treating wounds and ulcers, has been proposed by Dr. Jules Guyot. He published his views in the Archives Generales de Medicine, and afterwards he printed an extract from the Archives in the form of a pamphlet in 1835. The object of Dr. Guyot is simply to expose recent wounds of all descriptions, and ulcers, to hot and dry air, with the view of forcing a scab to form, by drying the clot and serum of a wound, or the pus of an ulcer. He made his first experiments on rabbits, on whom he inflicted several wounds, and afterwards placed the animals in a box having apertures, through which their heads projected. The air contained in these chambers was heated by a spirit lamp, generally to 25° of Fahrenheit, and sometimes higher. The animals were secured so that they could not move. Their wounds wept at first serum, but as they dried, their edges approached each other. In some cases, no tumefaction, nor appearance of inflammation was observed; in others suppuration took place after some days, underneath the crust; but by a longer exposure to the heated air, the pus thus formed, also dried ultimately into a thin scab. After it was removed, the wound was found to have been perfectly cicatrized underneath.

Dr. Guyot was not so successful in getting ulcers in the human subject to heal in this manner. After two or three weeks' trial, he was obliged in some cases to relinquish it, the patients not being able to bear the fatigue of having the limb so long confined to a box, without any change of position; nevertheless he did succeed in curing by the process of scabbing some ulcers of long standing and of an obstinate character, although pus formed again and again under the dried films which covered the ulcers. Dr. Guyot imputes great virtue to the heat, but it would seem to be merely instrumental to the drying of the serum, lymph, or pus, which may happen to lie on the wounds or ulcers." 208.

We do not anticipate much from this. But we must quit Dr. Macartney. We think our readers will agree with us that much instruction and more pleasure may be derived from a perusal of Dr. Macartney's volume. That gentleman has the good wishes of all on his retirement from the lecturer's chair.

